# Amino acid oxidation methods to determine amino acid requirements: do we require lengthy adaptation periods?

**DOI:** 10.1017/S0007114522002720

**Published:** 2023-06-14

**Authors:** Sylwia Szwiega, Paul B. Pencharz, Ronald O. Ball, Christopher Tomlinson, Rajavel Elango, Glenda Courtney-Martin

**Affiliations:** 1 Research Institute, Hospital for Sick Children, Toronto, ON M5G 1X8, Canada; 2 Departments of Nutritional Sciences, University of Toronto, Toronto, M5S 2Z9 ON, Canada; 3 Departments of Paediatrics, University of Toronto, Toronto, M5S 2Z9 ON, Canada; 4 Faculty of Kinesiology and Physical Education, University of Toronto, Toronto, ON M5S 2Z9, Canada; 5 Department of Agriculture, Food and Nutritional Science, University of Alberta, Edmonton, T6G 2P5 AL, Canada; 6 Department of Pediatrics, School of Population and Public Health, University of British Columbia, Vancouver, BC, Canada; 7 BC Children’s Hospital Research Institute, BC Children’s Hospital, Vancouver, V5Z 4H4 BC, Canada

**Keywords:** Amino acid requirement, Adaptation, Indicator amino acid oxidation, Amino acid oxidation

## Abstract

Determination of indispensable amino acid (IAA) requirements necessitates a range of intakes of the test IAA and monitoring of the physiological response. Short-term methods are the most feasible for studying multiple intake levels in the same individual. Carbon oxidation methods measure the excretion of ^13^CO_2_ in breath from a labelled amino acid (AA) in response to varying intakes of the test AA following a period of adaptation. However, the length of adaptation to each AA intake level has been a source of debate and disagreement among researchers. The assertion of the minimally invasive indicator amino acid oxidation (IAAO) technique is that IAA requirements can be estimated after only a few hours (8 h) of adaptation to each test AA intake, suggesting that adaptation occurs rapidly in response to dietary adjustments. On the contrary, the assertion of most other techniques is that 6–7 d of adaptation is required when determining IAA needs. It has even been argued that a minimum of two weeks is needed to achieve complete adaptation. This review explores evidence regarding AA oxidation methods and whether long periods of adaptation to test IAA levels are necessary when estimating IAA requirements. It was found that the consumption of experimental diets containing lower test IAA intake for greater than 7 d violates the terms of a successful adaptive response. While there is some evidence that short-term 8 h IAAO is not different among different test amino acid intakes up to 7 d, it is unclear whether it impacts assessment of IAA requirements.

An adequate supply of dietary protein and its constituent amino acids (AA), particularly the indispensable amino acids (IAA), is essential for maintaining cellular integrity and function, health and reproduction^(1)^. Therefore, a clear understanding of the dietary requirements for total protein intake and IAA is imperative across the life cycle.

We have previously reviewed different approaches to define individual IAA requirements^(2)^. Briefly, a range of intakes of the test IAA from deficient to excess; ideally, a minimum of three response data points above and three response data points below the predicted requirement should be assessed. With a range of intakes, a two-phase linear regression analysis can be applied to determine an average requirement or breakpoint estimate. These criteria are necessary for determining IAA requirements. Moreover, the concept of adaptation needs to be considered in relation to protein and IAA requirements^(3)^. Meaningful estimates of IAA requirements can only be defined after subjects have adapted to each test IAA intake level for an appropriate period. Waterlow and others^(4–6)^ described physiological adaptation as the achievement of a repeatable, steady-state response, following a dietary change, without adverse consequences. This has influenced the design of studies using the traditional nitrogen balance method, as well as the more recent AA oxidation methods using stable isotope tracers.

IAA requirements were determined by the nitrogen (N) balance method by measuring changes in nitrogen excretion, namely urea, in response to varying levels of IAA intake. N-balance requires an adaptation period of at least 7 d for each test IAA intake because of the time required for equilibration of the slow-changing large body urea pool^(7,8)^.

Amino acid oxidation methods are based on partitioning AA between protein synthesis and oxidation. The body cannot store free AA; therefore, AA in excess of that required for protein synthesis will be removed from the body through oxidation. Most AA-degrading enzymes have high Michaelis constants (Kms) and are therefore rarely saturated over a normal range of plasma and tissue AA concentrations^(5,9)^. In contrast, aminoacyl t-RNA synthetase, the enzyme that catalyses the amino acylation of the immediate precursors of proteins (tRNAs), has a fairly low Km and therefore is fully charged and operates at a high rate even when AA availability is low^(5,9,10)^. The combined activities of these enzymes, along with the lack of storage for free AA in the body and the short half-life of plasma free AA pools, have two major consequences. First, AA are more efficiently partitioned towards protein synthesis, which increases when AA availability is low. Second, the partitioning of AA towards protein synthesis or oxidation occurs rapidly^(5,9)^.

## Do amino acid oxidation methods require lengthy adaptation periods?

### Adaptation *v*. accommodation

The length of time required to achieve adaptation to the level of IAA intake to determine requirements using AA oxidation methods has been a source of continuing debate and disagreement among investigators for years^(5,11–14)^. For example, the assumption of the indicator amino acid oxidation (IAAO) technique is that IAA requirements can be estimated after 8 h of exposure to the test AA intake, suggesting that adaptation occurs rapidly in response to dietary intake adjustments^(15)^. On the contrary, other groups have incorporated a 6 to 7 d adaptation period into their IAAO^(16)^ and direct amino acid oxidation (DAAO)^(17)^ protocols when determining IAA requirements. Some have even argued that a minimum 2 week adaptation period is required to achieve complete adaptation^(18)^. However, such lengthy periods of adaptation are not practical for studying multiple intake levels in an individual subject thus, impacting a repeated measures study design. Furthermore, long periods of adaptation to very low or excessive intake as required by typical study designs may lead to adverse changes in body protein homeostasis. In addition, extended periods of such extreme diets could lead to an ‘accommodation’ rather than ‘adaptation’^(5)^. Accommodation describes the response to a dietary change that results in a significant loss of an important function such as growth, body composition, AA balance, organ or system functions^(4–6,19,20)^. When studying the nutritional needs of healthy individuals, accommodation is outside the limits of adaptation and must be considered in the design and interpretation of requirement studies. Studies have shown that when rats were fed very low protein intake or threonine-free diets for 14 d, there was a gradual decrease in the concentration and activity of threonine dehydratase in the liver that reached a lower steady state within 1 to 2 weeks^(21,22)^. This might seem like adaptation was reached after 1 to 2 weeks. However, this response was accompanied by a significant loss of weight, which is neither sustainable nor healthy, violating the terms of successful adaptation. In humans, there is a possible grey area where adaptation and accommodation cannot be distinguished, given the difficulty of measuring some endpoints related to loss of function over a short time span, such as body weight and lean body mass. However, using stable isotope tracers to measure changes in end-points related to protein and AA kinetics might help address this issue.

The influence of adaptation on L-(1–^13^C) leucine kinetics, balance and requirement was investigated in a long-term experiment in which adult men received 7, 14 or 30 mg·kg^–1^·d^–1^ leucine for 3 weeks^(17)^. Although IAA requirements cannot be determined using only three levels of intake, the investigators did so and found no discernible variation in the leucine requirement after 1 or 3 weeks of adaptation to the experimental diets^(17)^. However, at 7 and 14 mg·kg^–1^·d^–1^ intake levels, L-(1–^13^C) leucine oxidation and flux continued to decrease by the third week despite improvements in leucine balance, indicating a decrease in the rate of whole-body protein turnover^(17)^. It may be challenging to interpret the nutritional significance of these responses; however, a decrease in the rate of whole-body protein turnover may be an important indicator of an adverse physiological event^(17)^. Changes in the rate of whole-body protein turnover are often associated with changes in the turnover of skeletal muscle proteins^(23)^. Muscle tissue plays an important role in responding to trauma or infection; thus, lower muscle protein turnover might impair this function and compromise the ability to resist environmental stressors^(5,23)^. Thus, it might be surmised that the extended 3 week experiment at a very low leucine intake lead to an accommodation rather than adaptation response. Young and Marchini also disagreed with the idea of long-term adaptation (i.e. 3 weeks) because an accommodative response was observed in their experiments and they suggested that ‘the limits of adaptation … are reached over a relatively short timeframe’, such as 7 d^(5,24,25)^. As a result, Young *et al.* adopted a 7 d adaptation period for DAAO and subsequently IAAO studies of IAA requirements^(26)^. However, what is unclear is whether a 7 d adaptation period was adopted because it was assessed as the minimum time required for adaptation or because a shorter time frame (less than 7 d) was never tested by said group.

## Experimental evidence for short-term adaptation periods

In an earlier DAAO study, following 6 d on a lysine adequate diet, growing rats were fed experimental diets containing different levels of lysine intake (either deficient or excess) for 10 d. Oxidation studies were performed on days 1, 2, 3, 4, 5 and 10 of adaptation to the experimental diet^(27)^. When lysine oxidation was plotted against intake, a definite break in the oxidation curve appeared after day 3, and the breakpoint remained fairly constant between 100 and 110 mg lysine per day, indicating a steady state^(27)^. Moreover, when weight gain was plotted against intake, the breakpoint was 114 mg lysine per day, which was considered the requirement for optimal growth in rats. The authors concluded that the oxidation method can provide a means of estimating IAA requirements and that adaptation of the oxidative mechanisms in rats in response to dietary changes in AA intake is relatively rapid^(27)^. This study provided evidence that 3 d of adaptation to the level of AA intake was adequate. However, there are significant differences between the metabolisms of rodents and mammals, making it challenging to interpret how these findings might apply to human experiments^(8)^.

When the IAAO method was validated in piglets, Kim *et al.* demonstrated that the rate of release of ^14^CO_2_ from the oxidation of a radioactively labelled AA responded to the level of AA in the experimental diets within a few hours of consumption^(28,29)^. When IAAO was then applied to study IAA requirements in humans, one of the first steps was to investigate the effect of adaptation. In a study of healthy adult men, prior adaptation to either 4·2 or 14 mg·kg^–1^·d^–1^ phenylalanine resulted in a phenylalanine requirement that was numerically different (10·8 and 8·6 mg·kg^–1^·d^–1^, respectively) but not statistically different^(30)^. It is unclear if these requirements were indeed not different or whether significance could not be established because of the relatively small sample size and low statistical power^(30)^. In that same study, however, phenylalanine oxidation did not differ at 3, 6 or 9 d after a change in phenylalanine intake, indicating that adaptation had occurred at least 3 d after the dietary change^(30)^.

Subsequent to these experiments, the necessity for adapting subjects to the same level of protein intake for at least two days before the AA oxidation experiment was demonstrated^(31)^. This was supported by the findings from a more recent publication showing that habituation to different levels of protein intake introduced variation in the rate of oxidation of the indicator AA on the study day^(32)^. Both studies, however, examined two to three levels of protein intake and thus could not make any conclusions regarding what level of protein intake is appropriate for adaptation prior to the AA oxidation experiment. Though, using IAAO, the protein requirement was established to be 0·93 g·kg^–1^·d^–1^ in young adults^(33)^, and it was later judged that adaptation to a protein intake similar to this level would be most suitable for AA oxidation experiments. Moreover, a number of studies have directly determined protein, and AA requirements in populations consuming varying levels of habitual protein intake and found that it may have little effect on the estimation of protein and amino acid requirements. For example, within the nitrogen balance database analysed by Rand *et al.*, there were several studies conducted in countries with generally low levels of protein intakes. The authors concluded that there was no apparent influence of the habitual diets of subjects on any of the outcomes^(34)^. Kurpad *et al.* compared leucine kinetics in healthy and chronically undernourished Indian men and found no difference in leucine balance between the groups^(35,36)^. Flux was slightly higher in the undernourished group compared with the healthy; however, the authors suggested that this was due to differences in BMI. This same group also compared the lysine requirement in healthy and chronically undernourished Indian men and found the lysine requirement to be 50 % higher in the undernourished group^(37,38)^. However, there is a high prevalence of intestinal parasitic infections in these populations, and thus, another study was conducted where the lysine requirement was determined in chronically undernourished Indian men before and after being treated for intestinal parasitic infections^(39)^. These subjects had a 50 % higher lysine requirement before treatment, and once treated for the infection the requirement dropped to same value for healthy well-nourished adults^(37,39)^. The authors concluded that parasitic infections in this population is responsible for the higher lysine requirement observed in persons with chronic undernutrition rather than the effect of undernutrition or long-term ‘adaptation’ to low protein intakes^(39)^.

Based on the evidence from earlier human experiments^(30,40)^ and animal work^(28,29)^, it was judged that following adaptation to an adequate protein intake (1·0 g·kg^–1^·d^–1^) for 2 d, IAA requirements can be measured after a period of only 8 h of exposure to the test AA intake. These assumptions of the short 8 h IAAO method have been highly criticised^(3,12,13,15,41–45)^, stating that IAAO cannot determine IAA requirements, as it is unlikely that humans adapt to a new level of AA intake after only a few hours.

## An examination of the criticism against short-term adaptations

The criticisms against short-term adaptation arise from the concept of the metabolic demand model proposed by Millward and Rivers^(13,46)^. According to this model, the habitual level of protein intake creates a level of AA oxidative catabolism, and this rate of oxidation continues regardless of the actual acute intake^(18)^. A minimum requirement value can be defined only under conditions that minimise the adaptive component (i.e. minimise the oxidative losses of AA). A series of complex N-balance and short-term ^13^C-leucine balance studies have been conducted to verify and quantify this adaptive component^(13,18,47–49)^. However, from these studies, a number of intriguing issues and significant points have been raised, some of which are highly speculative. Perhaps the most significant caveat is that the protein requirement was not determined in any of these studies. In one of these studies Quevedo *et al*. described the time course of adaptation in N-balance, N-excretion, AA-balance and oxidation over a 14 d period following a reduction in protein intake from 1·82 to 0·77 g·kg^–1^·d^–1(48)^. The rate of leucine oxidation responded in a similar manner to N-excretion – leucine oxidation progressively decreased until day 7, with no further difference from day 7 to 14. Leucine balance, like N-balance, was positive at the higher protein intake but upon switching to the lower protein intake, became highly negative on day 3 and positive and constant from days 7 to 14^(48)^. However, all subjects lost weight, indicating that such data cannot be used to resolve the issue of the time required to achieve steady-state leucine oxidation or N-excretion to determine the IAA requirement in healthy humans.

It is challenging to understand why the achievement of steady-state leucine oxidation required a period of adaptation as long as N excretion in those experiments^(48)^. The rate of AA oxidation relies on the properties of oxidative enzymes, which allow a more rapid response to changes in dietary AA intake than the slow equilibration of the body urea pool^(7,8)^. For example, in another study using the ^13^C-leucine oxidation method, protein turnover and oxidation responded almost immediately, within 2 to 4 h, to dietary adjustments^(50)^. Nevertheless, proponents of a longer period of adaptation maintain that when determining the minimum requirement for an IAA, the body must adapt to low intakes, a process that requires at least two weeks or longer^(12,14,18,41)^. Thus, when oxidation is measured after only a few hours of exposure to the test diets (e.g. IAAO), oxidation, and therefore the IAA requirement, becomes a reflection of the habitual diet rather than the intended test AA level^(3,12,13,15,41–45)^; although the assertion has not been experimentally proven.

A comparison between the lysine requirement derived using 24 h DAAO (30 mg·kg^–1^·d^–1^)^(51,52)^ and that derived using short 8 h IAAO (37 mg·kg^–1^·d^–1^)^(53)^ concluded that major differences in the requirements between these studies are a consequence of incomplete adaptation^(13)^. In the IAAO study, generous levels of lysine (∼60 mg·kg^–1^·d^–1^) were fed throughout the experiment, apart from the oxidation study day when the lysine test levels were fed for only a few hours. The mean lysine requirements reported by the short-term IAAO method were higher than other estimates reported for lysine and were taken as evidence that the dietary design (i.e. feeding generous intakes prior to the study) had set the requirement that was being measured^(13)^.

The 24 h IAAO balance (IAAB) method was developed to address the limitations of the 8 h IAAO method by including measures of fed and fasted oxidation (i.e. 24 h) after 6 d of adaptation to test AA levels^(16)^. Thus, IAA requirement values from 24 h IAAB studies can be compared with those from 8 h IAAO studies. As shown in Table 1, the requirement values reported by 8 h IAAO are approximately 4 mg·kg^–1^·d^–1^ higher for lysine and threonine, 6 mg·kg^–1^·d^–1^ higher for phenylalanine and 2 mg·kg^–1^·d^–1^ lower for methionine when compared with those reported by longer adaptation studies^(16,37,53–61)^. These differences between estimates are smaller in value than the error associated with each individual estimate (i.e. the 95 % CI); therefore, they may not be statistically different. Although numerically, estimates obtained by the shorter 8 h IAAO method are approximately 20 % higher than those reported by the longer 24 h IAAB method, with the exception of methionine. Therefore, comparing estimates obtained by these two AA oxidation methods does not further our understanding of whether adaptation can be reached over an 8 h period and, on the contrary, whether adaptation for longer periods, such as 7 d, is successful and free from adverse events.

**Table 1.** Comparison of adult human amino acid requirements determined by short-term IAAO and 24 h IAAO/IAAB studies*

Amino acid	Study reference	Method (number of levels each participant was studied at / number of levels / total number of data points)	Length of adaptation to experimental diet	Breakpoint estimate (mg·kg^–1^·d^–1^)
Lysine	Kurpad *et al.*, 2001a^(37)^	IAAO (2/4/32)	8 d + 12 h fed	28·2
	Kurpad *et al.*, 2001a^(37)^	IAAB (2/4/32)	8 d + 24 h fasted-fed	29·7
	Kurpad *et al.*, 2002a^(16)^	IAAO (2/4/36)	6 d + 12 h fed	31
	Kurpad *et al.*, 2002a^(16)^	IAAB (2/4/36)	6 d + 24 h fasted-fed	31
	Kurpad *et al.*, 2002a^(16)^	IAAB (2/4/36)	20 d + 24h fasted-fed	31
	Zello *et al.*, 1993^(53)^	IAAO (6/7/42)	8 h	36·9
	Kriengsinyos *et al.*, 2002^(54)^	IAAO (6/6/60)	8 h	35
Methionine (with no cysteine)	Kurpad *et al.*, 2003^(55)^	IAAO (3/7/63)	6 d + 12 h fed	14
	Kurpad *et al.*, 2003^(55)^	IAAB (3/7/63)	6 d + 24 h fasted-fed	15
	Di Buono *et al.*, 2001^(56)^	IAAO (6/6/36)	8 h	12·6
Phenylalanine (with no tyrosine)	Kurpad *et al.*, 2006^(57)^	IAAO (2/8/64)	6 d + 12 h fed	36
	Kurpad *et al.*, 2006^(57)^	IAAB (2/8/64)	6 d + 24 h fasted-fed	37
	Hsu *et al.*, 2006^(58)^	IAAO (8/8/40)	8 h	42
Threonine	Borgonha *et al.*, 2002^(59)^	IAAB (1/3/15)	6 d + 24 h fasted-fed	15
	Borgonha *et al.*, 2002^(59)^	IAAB (1/3/15)	13 d + 24h fasted-fed	15
	Kurpad *et al.*, 2002b^(60)^	IAAO (3/6/48)	6 d + 12 h fed	15
	Kurpad *et al.*, 2002b^(60)^	IAAB (3/6/48)	6 d + 24 h fasted-fed	15
	Wilson *et al.*, 2000^(61)^	IAAO (6/7/36)	8 h	19

IAAO, indicator amino acid oxidation; IAAB, indicator amino acid balance; DAAB, direct amino acid balance; DAAO, direct amino acid oxidation; d, day; h, hour(s).

* This table was adapted from reference 62 with permission.

## Indicator amino acid oxidation and experiments to test longer term adaptation

To directly investigate whether oxidation of the indicator AA can adapt over a 6 to 9 d period, our group performed several IAAO studies in growing and adult pigs on different dietary interventions, either to changes in IAA intake or total protein intake^(19)^. In growing pigs, phenylalanine oxidation doubled when animals received a lysine-deficient diet and remained elevated and constant between days 2 and 6^(19)^. When given excess lysine, phenylalanine oxidation decreased and remained low and constant between days 2 and 6^(19)^. When the adult pigs were fed 50 %, 100 % and 200 % of their protein requirement for 9 d, steady rates of phenylalanine oxidation were reached by the first day and did not change thereafter^(19)^. Thus, oxidation of the indicator AA responds rapidly, within 1–2 d, to changes in the intake of total dietary protein or a single IAA. In addition, this quick response is valid, regardless of whether the animal is growing or at maintenance.

More recently, investigators of the IAAO method attempted to address criticisms of the 8 h minimally invasive protocol in humans^(62)^. After 2 d of adaptation to adequate protein intake, adult subjects were switched to diets containing 5, 20, 35 or 70 mg·kg^–1^·d^–1^ lysine for 7 d, and IAAO studies were performed after 8 h, 3 and 7 d^(62)^. The fractional rate of appearance of ^13^CO_2_ in breath from oxidation of the indicator AA (F^13^CO_2_) was not different across all lengths of adaptation to the diets^(62)^. The indicator oxidation was only affected by the level of lysine^(62)^. Since adaptation can be regarded as complete at the first time point of a steady-state plateau in a series of oxidation measurements, the authors concluded that the minimally invasive IAAO protocol involving 2 d of adaptation to adequate protein intake followed by an 8 h IAAO study, where subjects are adapted to the test AA level, is sufficient to estimate IAA requirements^(62)^. However, these conclusions have not been fully accepted by the scientific community because the lysine requirement was not directly determined.

### Conclusions: scientific and practical considerations for the length of adaptation

The two-phase linear regression is the preferred statistical model because it yields an objective and defendable estimate of nutrient requirement^(63,64)^. Therefore, six levels of AA intake should be carefully selected so that three or more levels fall above and below the estimated requirement to statistically satisfy two lines^(45)^. Finally, to control the large intra-individual variability, a repeated measures design is favoured, where each subject is studied over multiple levels of intake of the test AA^(2,65,66)^. A major limitation of the 24 h IAAB method is that each study day was approximately 24 h in length following a 6 d adaptation period. Thus, applying these practices would require that subjects follow strict, unpalatable, AA formula-based diets for 42 d when using a repeated measures design at six test levels. This is in contrast to the minimally invasive IAAO method, in which subjects only need to consume the diet for 8 h at each test level; thus, a total of 6 to 7 d is adequate in a repeated measures design. Therefore, a minimally invasive model is more practical, allowing for AA requirement estimates in all populations, including those who are vulnerable. This is of special importance since the most recent DRI report stated that a high priority should be given to research studies that help identify the requirements of IAA for all life stages and gender groups, most of which are considered vulnerable populations^(1)^.

While studies have shown that the indicator AA oxidation (measured as F^13^CO_2_) is not affected by 8 h, 3 or 7 d of adaptation to experimental diets^(62)^ and that AA oxidation responds quickly to dietary changes in AA intake^(19,27)^ the assertion that the requirement for an IAA can be determined after only a few hours of adaptation has not been fully accepted. This is because IAA requirements were not determined in studies assessing the effect of length of adaptation on IAAO responses, such as F^13^CO_2_^(19,62)^. Despite the higher IAA requirement estimates reported by studies using the shorter 8-h IAAO method compared with longer, 7 d, AA oxidation methods, these studies are often conducted in different populations with slight variations in study design. No study has directly compared the effect of increasing the length of adaptation on requirement estimates. Therefore, future research should aim to validate 8 h against 7 d of adaptation in an IAA requirement study. Reducing the length of adaptation would not only be ethically appropriate but would also assist in reducing study costs and subject burden and would be useful in studying vulnerable populations. Admittedly, the only approach to resolve this dilemma is to directly determine IAA requirements after varying the lengths of adaptation to specific IAA intake levels.
